# Detection of *Thelazia callipaeda* in *Phortica variegata* and spread of canine thelaziosis to new areas in Spain

**DOI:** 10.1186/s13071-018-2773-0

**Published:** 2018-03-20

**Authors:** Valentina Marino, Rosa Gálvez, Vito Colella, Juliana Sarquis, Rocío Checa, Ana Montoya, Juan P. Barrera, Sonia Domínguez, Riccardo Paolo Lia, Domenico Otranto, Guadalupe Miró

**Affiliations:** 1 Department of Animal Health, Faculty of Veterinary Medicine, Complutense University of Madrid, Madrid, Spain; 2 Department of Veterinary Medicine, University of Bari, Valenzano, Bari, Italy

**Keywords:** *Phortica variegata*, Flies, Vector, *Thelazia callipaeda*, Eyeworms, Autochthonous cases, PCR, Spain

## Abstract

**Background:**

The fruit fly *Phortica variegata* (Drosophilidae: Steganinae) feeds on the ocular secretions of animals and humans, and has been described as an intermediate host of the eye worm *Thelazia callipaeda* (Spirurida: Thelaziidae) in Italy. Despite the increased detection of *T. callipaeda* in many European countries, information about its vector role in natural conditions is still limited. In the Iberian Peninsula, thelaziosis caused by *T. callipaeda* has been reported in dogs, cats, red foxes, wild rabbits and humans.

**Methods:**

In the last seven years, we have detected increased numbers of cases of canine thelaziosis at three locations in mainland Spain: Site 1, La Vera region (Cáceres Province, central-western Spain; 51 cases); Site 2, El Escorial municipality (Madrid Community, central Spain; 23 cases); and Site 3, Miraflores de la Sierra municipality (Madrid Community, central Spain; 41 cases). Site 1 is considered endemic for *T. callipaeda* while the other two sites have been recently recognised as risk zones for *T. callipaeda* infection.

**Results:**

From June 2016 to September 2017, 2162 flies were collected and morphologically identified as *Phortica* spp. (Site 1, *n* = 395; Site 2, *n* = 1544; and Site 3, *n* = 223). Upon dissection, third-stage *T. callipaeda* larvae were found in two out of 155 flies examined from Site 1, and both these larvae tested molecularly positive for the eye worm. Of the 395 flies collected from Site 1, 371 were molecularly processed for arthropod species identification and *T. callipaeda* detection. All 371 flies were identified as *P. variegata* and 28 (7.5%; 95% CI: 4.8–10%) tested positive for *T. callipaeda* DNA haplotype 1.

**Conclusions:**

Our findings indicate that *T. callipaeda* circulates among dogs and *P. variegata* in Spain, where zoonotic cases have been also reported. The co-existence of canine thelaziosis and *Phortica* spp. in geographical areas previously considered free of the eye worm indicates a risk of infection for both animals and humans living in this region.

## Background

*Thelazia callipaeda*, Railliet & Henry, 1910 (Spirurida: Thelaziidae) is a nematode that causes eye infection in several mammals, including humans. In their definitive hosts, adult parasites are responsible for subclinical to clinical ocular thelaziosis [1]. The clinical picture produced is mostly the outcome of mechanical damage to the conjunctival and corneal epithelium caused by the eye worm’s serrated cuticle, and of the burden of adult nematodes inhabiting the conjunctival sac [1]. The most common clinical signs are conjunctivitis followed by lachrymal discharge, epiphora and in severe cases, keratitis, corneal opacity or ulcers [1].

*Thelazia callipaeda* infection has been described not only in dogs and cats but also in red foxes (*Vulpes vulpes*), gray wolves (*Canis lupus*), beech martens (*Martes foina*), brown hares (*Lepus europaeus*) and wild cats (*Felis silvestris*) [2]. In the Iberian Peninsula, thelaziosis caused by *T. callipaeda* has been detected in red foxes [3, 4] and wild rabbits [5].

Based on initial reports and the high number of cases of *T. callipaeda* in Asia [6, 7], the parasite is known as “the oriental eye worm”. In Asian countries, human thelaziosis is considered a neglected zoonotic disease due to the high number of cases reported amongst people living in poor and rural areas [8]. In Europe, cases of human thelaziosis have been described in Italy and France [9], Spain [10–12], and more recently, in Croatia and Serbia [13, 14].

In Spain, the first autochthonous case of ocular thelaziosis was reported in 2010 in a dog that had spent a few weeks in the region of La Vera (Cáceres Province, western Spain) [15]. Following further reported cases of canine thelaziosis in this region [16], the geographical area is now considered endemic for canine thelaziosis. Prevalences recorded in dogs of La Vera and bordering areas (provinces of Salamanca, Ávila and Toledo) have been estimated at around 40% [17, 18]. Of note, in 2012 the first case of feline thelaziosis was detected in La Vera [19].

The distributional range of this nematode infection is thought to be related to that of its vector and intermediate host, *Phortica variegata* Fallén, 1823 (Drosophilidae: Steganinae) [20–22]. This non-biting fruit fly usually feeds on fermenting fruits and other vegetables, though males display zoophilic behaviour [21]. *Phortica variegata* and *Phortica okadai* Okada, 1956 have been described as vectors of *T. callipaeda* in Europe and China, respectively [20, 22]. Under experimental conditions, both males and females can act as vectors of *T. callipaeda* [20], but in natural conditions, only males have been observed to transmit infective third-stage larvae (L3) to the definitive host [21]. To date, reports exist of *P. variegata* acting as intermediate host and vector of *T. callipaeda* under natural conditions only in Italy, although the fruit fly has also been detected in other European countries where autochthonous infections of *T. callipaeda* have been reported [17, 23].

However, while *P. variegata* is the main vector candidate for *T. callipaeda* in European countries, others species like *Phortica semivirgo* Máca, 1977 are thought to play a role in the transmission of this eye worm [24]. Furthermore, owing to the similar taxonomic characters of adult worms of *P. variegata* and *P. semivirgo*, identification based only on morphology is difficult and requires specific technical skills (reviewed in [25]).

In this paper, we describe the detection of *P. variegata* specimens naturally infected with *T. callipaeda* in Spain. In addition, we report 115 new cases of canine thelaziosis in three locations of Spain, and assess the seasonal distribution of *Phortica* spp. in these areas.

## Methods

### Sampling sites

Flies were collected in three different areas of the Iberian Peninsula where cases of thelaziosis in dogs and cats have been reported. The collection sites were designated as:

Site 1. La Vera region (northern Cáceres Province); central-western Spain (40°9'41"N, 5°23'13"W); altitude 472 m above sea level (masl);

Site 2. El Escorial municipality (northwest Madrid Community); Sierra de Guadarrama (40°36'10"N, 4°7'22"W); altitude 946 masl;

Site 3. Miraflores de la Sierra municipality (northwest Madrid Community); Sierra de Guadarrama (40°48'54"N, 3°46'15"W); altitude 1147 masl.

At the three study sites, climate and vegetation are typically Mediterranean. Thus, summers are hot and dry, and maximum rainfall is recorded in autumn and spring. The vegetation in these mountainous regions consists of pines (*Pinus brutia*) and holm oaks (*Quercus ilex)* in lower areas, and pasture and shrublands in high mountain areas. La Vera region borders with land given over to fruit production (apples, pears, figs, blueberries, raspberries and vineyards). At the three sites, mammals such as deer (*Cervus elaphus*), wild boar (*Sus scrofa*), roe deer (*Capreolus capreolus*), fallow deer (*Dama dama)*, badgers (*Meles meles*), mustelids, wild cats (*Felis silvestris*), red foxes (*Vulpes vulpes*), gray wolves (*Canis lupus*) and hares (*Lepus europaeus*) abound [26, 27].

### Climate data collection

Temperatures (°C) and relative humidity, RH (%), were recorded using a thermohygrometer (PCE-MHT 1, PCE Instruments, Southampton, UK). Each sampling site was also assigned macroclimate variables (maximum, minimum and average daily temperature, average temperature and precipitation of the current month, and wind speed) provided by the Spanish Meteorological Agency (AEMet) for the closest meteorological station [28].

### Diagnosis of thelaziosis in dogs

All surveyed dogs were subjected to ocular examination after the administration of anaesthetic eye drops (tetracaine hydrochloride and naphazoline hydrochloride). *Thelazia callipaeda* eye worms were collected from the conjunctival sac of infected dogs using sterile cotton swabs or by flushing with physiological saline solution. In total, 287 dogs were examined at the three sites. At Site 1, where a prevalence of 40% had been previously reported for this thelaziosis [17], a population of 75 hunting dogs living outdoors was selected and classified as consisting of “high-risk owned dogs”. At Sites 2 and 3, where veterinarians had reported cases of thelaziosis both in dogs and cats (Guadalupe Miró, unpublished observations), 88 and 124 owned dogs were actively sampled, respectively. Animals sampled at Sites 2 and 3 were pets classified as “low-risk owned dogs”.

### Fly collection, processing and morphological identification

**Fig. 1:**
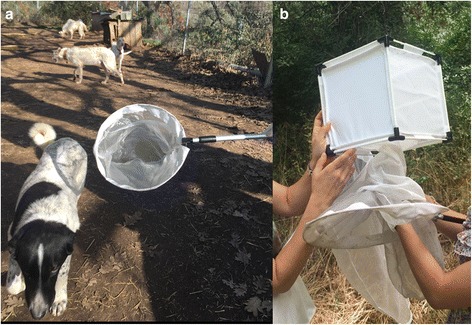
**a** Netting procedure. **b** Cage (20 × 20 × 20 cm) used to store the *Phortica* flies

Non-biting flies were netted with a butterfly net around the eyes of dogs and humans (Fig. 1a). Although trapping is a valid technique, it is not specific and large numbers of other drosophilids may be captured. While time-consuming, netting is a cheap and ready-to-use method.

**Fig. 2:**
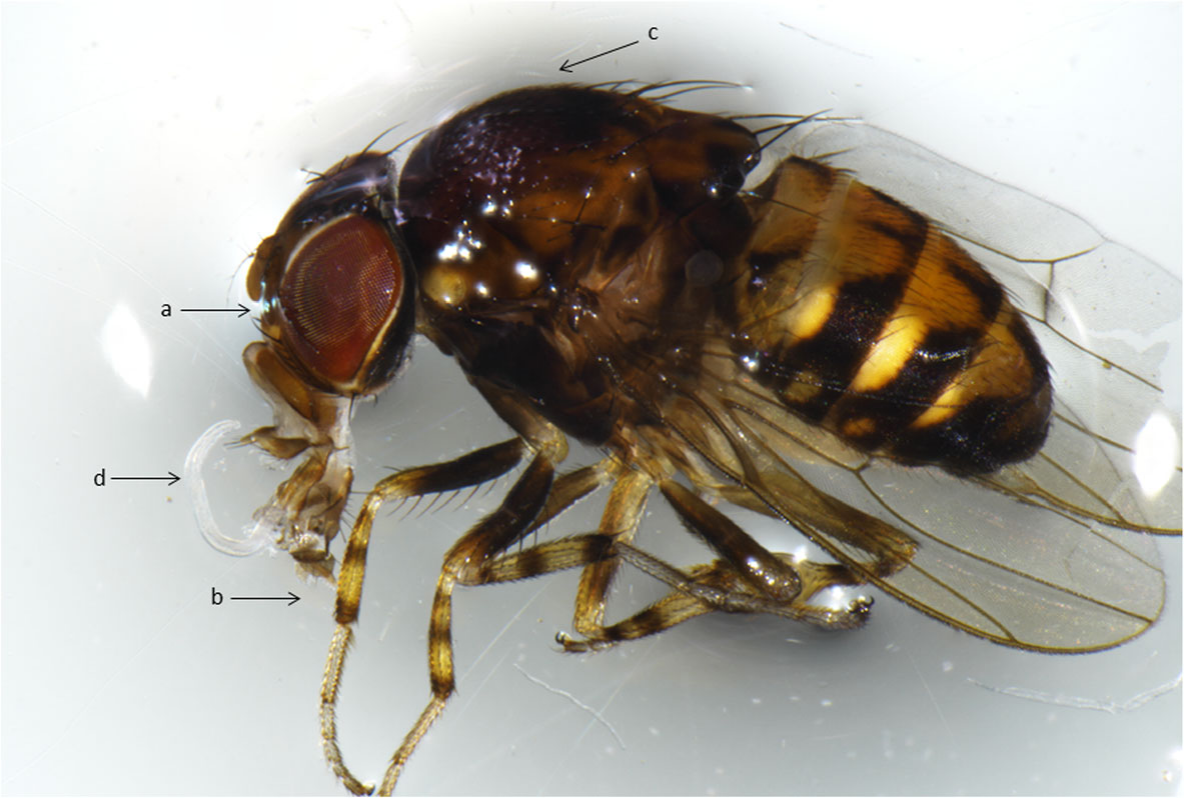
Male *P. variegata*, lateral view, showing: **a** pale ring around the eyes; **b** yellow tibiae with three dark bands; **c** grey spots on the thorax and **d** third-stage *T. callipaeda* larva in the proboscis

Flies were captured over a period of about 1 h, usually in the morning. Captured flies were stored in a 20 × 20 × 20 cm cage made from a 0.4 mm^2^ pore-size mesh (Fig. 1b) and transferred to our laboratory at the Department of Animal Health (Faculty of Veterinary Medicine, Complutense University of Madrid) for identification (Fig. 2). The density of flies was calculated as the number of specimens captured over the recorded sampling time.

To detect the presence of L3 *T. callipaeda* larvae in the flies’ proboscis, collected specimens of *Phortica* spp. were examined by dissection and visual inspection. Only live flies were dissected in a drop of physiological saline. Dead specimens were stored individually in 70% ethanol. Morphologically identified *Phortica* spp. flies from Site 1 were also subjected to molecular analyses. All flies (dissected or not) were sexed according to the morphology of the terminal part of the abdomen and stored in 70% ethanol in individual vials for further analyses.

Morphological identification was performed under a stereomicroscope (Stemi DV4®, Carl Zeiss, Göttingen, Germany) on the basis of taxonomic characters, such as a pale ring around the eyes, yellow tibiae with three dark bands and gray spots on the thorax (Fig. 2) [25, 29]. The unequivocal identification of the species *P. variegata* and *P. semivirgo* is challenging and relies upon the appearance of the terminalia of males and females [25, 29]. The features looked for are the number of sensilla on each medial and dorsal branch of the anterior parameres of the genitalia (3 on each branch for *P. variegata*, 3–5 for P*. semivirgo*) and the pale ring around the eyes (brownish around the lower half of the eye for *P. variegata*, pale yellow along the whole eye margin for *P. semivirgo*). Some authors have also described intermediate forms in terms of morphological characters for these two species [23]. The proboscis was dissected to retrieve infective *T. callipaeda* larvae, and the abdomen, in particular the terminalia of males, to retrieve different larval stages of *T. callipaeda*. The number and location of larvae in the body of flies were recorded and the larvae were also identified using morphological keys [20, 30]. Collected *T. callipaeda* larvae were measured and photographed.

### DNA extraction, PCR and sequencing

Genomic DNA was extracted from individual flies (*n* = 371) and from nematodes (*n* = 2) isolated from two flies, using a commercial kit (DNeasy Blood & Tissue Kit, Qiagen, Hilden, Germany). A partial sequence of the mitochondrial cytochrome *c* oxidase subunit 1 gene (*cox*1, ~689 bp) was amplified by PCR for the detection of *T. callipaeda* DNA as described in a previous study [31]. To confirm the identification of the flies as *P. variegata*, specimens collected from Site 1 (*n* = 371/395) were subjected to PCR and sequencing, as described elsewhere [32]. In brief, amplicons were purified using Ultrafree-DA columns (Amicon, Millipore, Bedford, MA, USA) and sequenced directly with the Taq DyeDeoxyTerminator Cycle Sequencing Kit (v.2, Applied Biosystems, Foster, California, USA) in an automated sequencer (ABI-PRISM 377, Applied Biosystems). Sequences were aligned using the Geneious R9 software package [33] and compared (BLASTn) with those available in GenBank [34].

### Statistical analysis

Fly densities were recorded as the number of flies collected in one hour. A descriptive analysis of the main numerical variables recorded (temperature, relative humidity, rainfall, wind speed, density of flies and percentage of males) was performed using mean and standard deviation (SD) or medians and quartile ranges for quantitative variables. Fly density and male percentage were assigned as the dependent variables. Interaction between these two dependent variables and the climate data (independent quantitative variables) was assessed using Spearman’s correlation coefficient. For this analysis we used the SPSS 22 statistics package for Windows. Significance was set at *P* ≤ 0.05.

## Results

### Dogs

Of the 287 dogs examined at the three sites, 115 (40.1%, 95% CI: 34.6–45.8%) were found to be infected by *T. callipaeda.* At Sites 1, 2 and 3, the numbers of infected autochthonous dogs were 51 of 75 (68%, 95% CI: 56.8–77.5%), 23 of 88 (26.1%, 95% CI: 18.1–36.2%) and 41 of 124 (33.1%, 95% CI: 25.4–41.7%), respectively. There were significant differences in prevalence between the groups of dogs from the three sites (*χ*^2^ = 34.012, *df* = 2, *P* < 0.05).

Most infected animals presented ocular signs (*n* = 69; 60%) whereas no clinical signs were observed in 46 dogs (40%). The clinical signs most frequently observed were conjunctivitis (*n* = 59; 85.5%), petechiae and oedema (*n* = 4; 5.8%), keratitis (*n* = 3; 4.3%) and epiphora (*n* = 3; 4.3%).

### Fly collection and seasonal survey

In total, 2162 flies (1722 males and 489 females) were collected at the three sites surveyed (Tables 1, 2 and 3). All flies were morphologically identified as belonging to the genus *Phortica*. Climate data and densities of *Phortica* spp. captured at Sites 1, 2 and 3 are reported in Tables 1, 2 and 3, respectively.

**Table 1:** Density of *Phortica* spp. captured (*n* = 395) in 2017 at Site 1 (La Vera) and climate variables

Month	Microclimate variables		Macroclimate variables					Fly density (flies captured per h)	
	T_set_	RH_set_	T_max_ daily	T_min_ daily	T_mean_ daily	T_mean_ monthly	Rainfall monthly (mm)	Males	Females
May 2017	32.9	24.3	32.0	17.0	24.5	19.8	16.4	11	0.5
June 2017	33.8	28.8	35.7	21.0	28.4	25.7	12.8	33	0
July 2017	33.6	24.1	35.0	18.0	26.5	26.6	38.0	113.3	3.3
August 2017	26.7	43	27.9	13.6	20.8	27.2	48.0	92	3
September 2017	32.8	29.8	31.4	17.0	24.2	23.1	0	84	8

*Abbreviations*: *T* temperature (°C), *RH* relative humidity (%), *T*_*set*_ Temperature recorded at the time of capture, *RH*_*set*_ relative humidity recorded at the time of capture, *T*_*max*_ maximum temperature, *T*_*min*_ minimum temperature, *T*_*mean*_ mean temperature

**Table 2:** Density of *Phortica* spp. captured (*n* = 1544) at Site 2 (El Escorial) and climate variables

Month	Microclimate variables		Macroclimate variables					Fly density (flies captured per h)	
	T_set_	RH_set_	T_max_ daily	T_min_ daily	T_mean_ daily	T_mean_ monthly	Rainfall monthly (mm)	Males	Females
June 2016	–	–	30.9	13.6	22.3	21.3	40.0	44.0	0.4
June 2016^a^	–	–	35.1	19.6	27.3	21.3	40.0	86.0	3.5
July 2016	–	–	29.3	10.7	20	24.0	34.8	91.5	0
August 2016	28.4	31.7	33.7	18.4	26.1	26.0	3.6	137.2	2.0
September 2016	25.2	24.4	27.7	14.1	20.9	21.2	14.8	56.5	60.5
October 2016	26.2	26.9	25.0	10.6	17.8	16.1	276.0	43.2	74.4
October 2016^a^	22.0	29.8	23.5	8.9	16.2	16.1	276.0	13.3	27.3
November 2016	–	–	13.8	3.7	8.8	8.6	186.8	0	0
April 2017	–	–	18.8	4.8	11.8	14.3	32.4	5.3	0
May 2017	29.0	32.4	30.8	17.2	24.0	17.9	29.4	10.4	0
June 2017	26.3	32.1	26.9	15.8	21.3	24.2	31.2	33.3	0
July 2017	26.2	25.2	30.3	15.1	22.7	24.6	188.6	16.0	0
August 2017	28.2	35.3	32.2	17.5	24.9	24.5	49.4	112.0	2.0
September 2017	24.0	23.4	24.9	16.6	20.8	20.3	0	38.0	16.0

^a^More than one sampling in the same month

*Abbreviations*: *T* temperature (°C), *RH* relative humidity (%), *T*_*set*_ temperature recorded at the time of capture, *RH*_*set*_ relative humidity recorded at the time of capture, *T*_*max*_ maximum temperature, *T*_*min*_ minimum temperature, *T*_*mean*_ mean temperature

**Table 3:** Density of *Phortica* spp. captured (*n* = 223) at Site 3 (Miraflores de la Sierra) and climate variables

Month	Microclimate variables		Macroclimate variables					Fly density (flies captured per h)	
	T_set_	RH_set_	T_max_ daily	T_min_ daily	T_mean_ daily	T_mean_ monthly	Rainfall monthly (mm)	Males	Females
September 2016	23.3	24.4	25.4	3.9	14.6	16.4	16.4	46	26
June 2017	30.0	24.5	28.5	5.2	16.8	19.2	59.6	10	0
July 2017	26.3	36.9	30.8	9.0	19.9	19.5	85.6	66	1
August 2017	–	–	31.2	9.0	20.1	19.5	132.4	6	0
September 2017	–	–	24.7	2.8	13.8	15.0	9.6	4	4

*Abbreviations*: *T* temperature (°C), *RH* relative humidity (%), *T*_*set*_ temperature recorded at the time of capture, *RH*_*set*_ relative humidity recorded at the time of capture, *T*_*max*_ maximum temperature, *T*_*min*_ minimum temperature, *T*_*mean*_ mean temperature

The numbers of *Phortica* spp. flies captured at each site over different sampling periods (given in parentheses) were: Site 1 (May-September 2017, *n* = 395); Site 2 (total *n* = 1544; June-November 2016, *n* = 1410; April-September 2017, *n* = 134); and Site 3 (total *n* = 223; September 2016, *n* = 144; June-September 2017, *n* = 79).

**Fig. 3:**
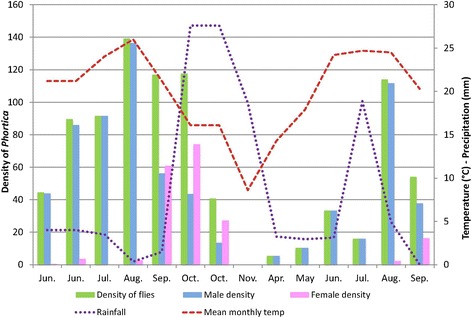
Seasonal trends in *Phortica variegata* fly densities (Site 2)

Overall, *Phortica* spp. specimens were found in peri-urban areas characterised by a maximum daily temperature of 35.7 °C (Site 1) and a minimum daily temperature of 2.8 °C (Site 3). The lowest average daily temperature recorded was 11.8 °C in April 2017 for Site 2; in these conditions an average of 5.3 flies/h could be collected. No flies were collected when the average daily temperature was 8.8 °C (November 2016). The highest numbers of *Phortica* spp. were recorded in August at Site 2, when the average monthly temperature was higher (26 °C in 2016 and 24.5 °C in 2017) (Fig. 3). In all places, the total percentage of male *Phortica* flies (79.65%) was significantly higher than females (20.35%).

At Site 1, numbers of captured *Phortica* spp. peaked in July, August and September; 96% of specimens being male (*n* = 380/395). At Site 2, 74.4% (*n* = 1049/1410) of *Phortica* spp. captured in 2016 were males, and females were increasingly collected in September and October of this year. In 2017, the proportion of male flies captured at this site was 93.3% (*n* = 125/134) (Fig. 3). At Site 3, the percentage of male flies collected in September 2016 was 63.2% (*n* = 92/144). The percentage of male flies collected from June to September the following year (2017) was 96.2% (*n* =76/79).

### Bivariate analysis

Positive moderate correlation was observed between the percentage of male fly specimens collected and the temperature recorded at the time of capture (*r*_(17)_ = 0.53, *P* = 0.02). Fly density was also positively correlated with minimum and mean daily temperature, respectively (*r*_(24)_ = 0.45, *P* = 0.02 and *r*_(24)_ = 0.42, *P* = 0.04), and with the mean temperature of the current month, although this time without significance (*r*_(24)_ = 0.50, *P* = 0.11). Fly density and the percentage of male flies showed negative correlation (*r*_(23)_ = -0.44, *P* = 0.03). No correlations emerged between neither of the dependent variables (fly density and percentage males) and RH at the time of capture, maximum daily temperature, average monthly rainfall and wind speed (Table 4).

**Table 4:** Climate variables and bivariate factors related to fly density and the percentage of males. Values of coefficient (*r*) and *P*-values obtained by Spearman’s correlation analysis

	Fly density	% males	T_set_	RH_set_	T_max_ daily	T_min_ daily	T_mean_ daily	T_mean_ monthly	Wind speed	Rainfall monthly
Fly density	*r*_(23)_ = 1	*r*_(23)_ = -0.44 *P* = 0.03*	*r*_(17)_ = -0.18 *P* = 0.4	*r*_(17)_ = 0.07 *P* = 0.7	*r*_(24)_ = 0.33 *P* = 0.11	*r*_(24)_ = 0.45 *P* = 0.02*	r_(24)_ = 0.42 *P* = 0.04*	*r*_(24)_ = 0.50 *P* = 0.11	*r*_(22)_ = 0.27 *P* = 0.22	*r*_(21)_ = -0.16 *P* = 0.47
% Males	*r*_(23)_ = -0.44 *P* = 0.03*	*r*_(23)_ = 1	*r*_(17)_ = 0.53 *P* = 0.02*	*r*_(17)_ = 0.3 *P* = 0.2	*r*_(23)_ = 0.30 *P* = 0.15	*r*_(23)_ = 0.14 *P* = 0.49	*r*_(23)_ = 0.18 *P* = 0.38	*r*_(23)_ = 0.15 *P* = 0.49	*r*_(21)_ = 0.36 *P* = 0.11	*r*_(20)_ = 0.1 *P* = 0.64

*P < 0.05

*Abbreviations*: *T*_*set*_ temperature recorded at the time of capture, *RH*_*set*_ relative humidity recorded at the time of capture, *T*_*max*_ maximum temperature, *T*_*min*_ minimum temperature, *T*_*mean*_ mean temperature

### Detection of *Thelazia callipaeda* in *Phortica variegata*

The numbers of flies that were still alive at the time of dissection were 155 (39.2%), 1088 (70.5%) and 202 (90.6%) for Sites 1, 2 and 3, respectively. Upon dissection, one *T. callipaeda* L3 was detected in each proboscis of two flies collected at Site 1 (Fig. 2), while no flies from Sites 2 and 3 were found to be infected by *T. callipaeda*.

Twenty eight of 371 *Phortica* flies from Site 1 that were molecularly processed (7.5%; 95% CI: 4.8–10%) returned a positive result for *T. callipaeda* with sequences 100% identical to those of *T. callipaeda* haplotype 1 (GenBank: AM042549) and to those obtained for the L3 larvae detected (*n* = 2; Fig. 2, Table 5). Insect nucleotide sequences of flies scoring positive for *T. callipaeda* DNA (*n* = 28) were 99–100% identical to those of *P. variegata* (GenBank: EF576934).

**Table 5:** Number, sex and positive *Phortica* spp. from Site 1 dissected between May and September 2017

Month	No. of flies		Dissection		Molecular analysis	
			Examined (infected) flies		Examined (infected) flies	
	M	F	M	F	M	F
May	22	1	14 (0)	1 (0)	9 (1)	0 (0)
June	33	0	13 (1)	0 (0)	33 (2)	0 (0)
July	170	5	25 (0)	5 (0)	165 (11)	1 (0)
August	92	3	50 (1)	3 (0)	92 (14)	3 (0)
September	63	6	42 (0)	2 (0)	62 (0)	6 (0)
Total	380	15	144 (2)	11 (0)	361 (28)	10 (0)

*Abbreviations*: *F* female, *M* male

Of the 28 infected flies, one was captured in May (*n* = 1/9; 11.11%), 2 in June (*n* = 2/33; 6.06%), 11 in July (*n* = 11/166; 6.63%) and 14 in August (*n* = 14/95; 14.74%; Table 5).

## Discussion

In this study, the detection of *T. callipaeda* in *P. variegata* and its potential vectorial role have been described for the first time in Spain*.* In addition, we report 115 new cases of canine thelaziosis at three localities in Spain, and assess the seasonal distribution of *Phortica* spp. in these areas, thus confirming that this nematode circulates among dogs and flies. The percentage of infected dogs showing clinical signs (i.e. 60%) was considerably higher than that recorded in previous studies (15.4%) [17]. This finding is probably related to the fact that dogs were examined during late stages of infection.

The high prevalence of *T. callipaeda* infection in the dogs from Site 1 (La Vera) was as expected, though still higher (51 out of 75, 68%) than in an earlier report (i.e. 182/456, 40%) [17]. In contrast, the infection prevalences recorded at the other two sites in Madrid, seven years after the detection of the first infection focus (La Vera), were unexpected, as the affected dogs had not travelled to endemic areas. Indeed, these cases represent new areas of expansion of the parasite within a few years. Differences in prevalence between the groups of dogs from the three sites studied were statistically significant and it may be due to the fact that the infection has been established in Sites 2 and 3 only in the last few years. In addition, dogs from the Site 1 were living outdoors and were selected and classified as consisting of “high-risk owned dogs”. Although different animal species (such as red foxes, gray wolves, etc.) have been reported in the three sites, red foxes positive to *Thelazia* spp. have already been recorded only in Site 1 [3]; this may be associated with the higher prevalence of infection at this site.

Furthermore, the co-occurrence of canine thelaziosis and *Phortica* spp. in areas previously considered free of the parasite determines a risk of infection for animals and humans living in this region, and suggests the likely spread of *T. callipaeda* among dogs in Spain. To the best of our knowledge, reports so far of canine thelaziosis in the Madrid Community only amounted to imported cases from the La Vera region. These new findings indicate a need to now consider these areas of central Spain as autochthonous for canine thelaziosis.

The first report of *T. callipaeda* in *P. variegata* was published in 1963 by Kozlov who described larvae in the proboscis of these fruit flies [21]. In 2002 and 2005, *P. okadaki* and *P. variegata* were described as vectors of *T. callipaeda* in China and southern Europe, respectively [20, 22]. In 2006, *P. variegata* was cited as an intermediate host of *T. callipaeda* [21].

An increase in the occurrence of *T. callipaeda* in western and eastern Europe has been recently described [35]. In Spain, new cases of thelaziosis are steadily rising in different parts of the country [36], though up until now, the occurrence of *T. callipaeda* in *P. variegata* was reported only in Italy [21].

Our collection sites of *Phortica* spp. are at the same latitude as other European countries and China where thelaziosis is endemic in the range 39–46° North, where the dominant vegetation has also been previously associated with the presence of *Phortica* flies [23, 37–45].

The sex ratio of the *Phortica* flies was male-oriented, overall proportions of male flies (1722/2162, 79.6%) being significantly higher than female flies (*r*_(23)_ = -0.44, *P* = 0.03). This predominance of males found around the eyes may be explained by dietary habits (these insects supplement their protein intake for gonadotrophic development; reviewed by Otranto et al. [21]). According to a previous survey [23], by netting around the eyes of dogs, 79.6% of all 2162 *Phortica* collected were males, and there were only a few females, most of them captured at the end of the summer. Contrary to the results obtained by other authors [21], not all of the present flies collected around the eyes were male. *Phortica* flies feed on ocular secretions mainly in the second half of the season (July-October), which may be because of dietary needs or because of the higher abundance and activity of *Phortica* males in these months [21]. As the total number of flies increases at the end of summer, so does the overall number of females. Notwithstanding the previous studies, there is still a lack of knowledge about the behaviour of these flies especially regarding their feeding requirements. This information may be crucial for the design of preventative measures such as repellent formulations targeted at avoiding the feeding of *Phortica* vectors on infected/uninfected animals.

The length of the activity period of *Phortica* spp. is largely conditioned by climate conditions. Flies were detected from April when average daily temperatures were 11.8 °C and the average monthly temperature was 14.3 °C. The number of flies increased with rising temperatures, peaking in August. Finally, at Site 2 the last specimens were captured in September, in line with previous reports [21, 23]. Peaks of *P. variegata* were recorded in mid-summer (August), with temperature being positively correlated with fly density.

By correlating *Phortica* spp. fly densities with *T. callipaeda* prevalences in dogs, vector densities may be then used to assess the presence of canine thelaziosis in a given area. The prevalence of naturally infected *P. variegata* reported here (i.e. up to 14.7% in August; 14 out of 95 flies captured) is higher than that recorded by Kozlov in 1963 (1.36%) [21] and more recently (2006) by Otranto (1.34%) [21]. We consider that such a high percentage of infected flies could represent a threat, leading to the spread of this parasitosis in Spain. Further studies are needed to confirm this idea and address its consequences.

The molecular approach described in this paper is reliable for detecting species of *Thelazia* in their different vectors avoiding frequent constraints (e.g. time-consuming procedures, operator expertise, sensitivity of the methodology, misidentification). We used this approach to investigate the role of flies as vectors along with fly dissection and morphological identification of larval stages. In effect, the detection of parasite DNA in arthropod hosts alone is not sufficient proof of their vectorial role or the part played in the ecology of a given vector borne disease. This is because it is not possible to differentiate and even describe larval states in the case of positive results. The molecular characterization of the nematodes examined in the present study served to confirm the identical nature of all *cox*1 sequences to those of *T. callipaeda* haplotype 1. This haplotype has been detected in humans and in domestic animals in Europe [20].

In our study, only *P. variegata* males were found to be infected by *T. callipaeda*, thus confirming the hypothesis that only males act as intermediate host under natural conditions [21]. Unlike other drosophilid species, *P. variegata* feeds on the lachrymal secretions of humans and carnivores [25]. The detection of *T. callipaeda* exclusively in male specimens of its arthropod vector is of interest from both parasitological and ecological standpoints, and represents a unique case in which a male drosophilid fly feeding on vertebrate host secretions can transmit a vector-borne pathogen under natural conditions [46].

## Conclusions

We here confirm the occurrence of *P. variegata* in Spain and its potential vectorial role of *T. callipaeda*, posing a threat to animals and humans. The results of this study serve to fill gaps in the knowledge of the biological cycle of *T. callipaeda* in southern Europe and offer new prospects for epidemiological studies on thelaziosis and for the design of appropriate control measures. In addition, autochthonous cases of canine thelaziosis are reported for the first time for new areas in Spain.

## Abbreviations

AEMet: Spanish Meteorological Agency
L3: Third-stage larvae
masl: Meters above sea level
SD: Standard deviation
